# Object Detection in Adverse Weather for Autonomous Driving through Data Merging and YOLOv8

**DOI:** 10.3390/s23208471

**Published:** 2023-10-14

**Authors:** Debasis Kumar, Naveed Muhammad

**Affiliations:** Institute of Computer Science, University of Tartu, Narva Maantee 18, 51009 Tartu, Estonia; naveed.muhammad@ut.ee

**Keywords:** autonomous driving, harsh weather, object detection, data merging, deep neural networks, YOLOv8

## Abstract

For autonomous driving, perception is a primary and essential element that fundamentally deals with the insight into the ego vehicle’s environment through sensors. Perception is challenging, wherein it suffers from dynamic objects and continuous environmental changes. The issue grows worse due to interrupting the quality of perception via adverse weather such as snow, rain, fog, night light, sand storms, strong daylight, etc. In this work, we have tried to improve camera-based perception accuracy, such as autonomous-driving-related object detection in adverse weather. We proposed the improvement of YOLOv8-based object detection in adverse weather through transfer learning using merged data from various harsh weather datasets. Two prosperous open-source datasets (ACDC and DAWN) and their merged dataset were used to detect primary objects on the road in harsh weather. A set of training weights was collected from training on the individual datasets, their merged versions, and several subsets of those datasets according to their characteristics. A comparison between the training weights also occurred by evaluating the detection performance on the datasets mentioned earlier and their subsets. The evaluation revealed that using custom datasets for training significantly improved the detection performance compared to the YOLOv8 base weights. Furthermore, using more images through the feature-related data merging technique steadily increased the object detection performance.

## 1. Introduction

Autonomous driving has promised many benefits for society, with the most important being safe transportation. The rapid development of computing technology and the low-cost manufacturing of sensors have significantly impacted autonomous driving research. An autonomous vehicle’s autonomy is divided into six levels based on the level of human involvement during its operation [1]. It is inescapably reliant on autonomy subsystems such as perception, localization, behavior prediction, planning, control, etc. Among these, perception is a vital component of autonomous driving that deals with understanding the ego vehicle’s environment using sensors. Then, the perception result helps to execute consequent tasks through this environmental information. Due to dynamic objects and ongoing environmental changes, perception can be challenging. Moreover, the quality of perception becomes worse as a result of interruptions in the perception quality caused by inclement weather such as snow, rain, fog, night light, sand storms, etc. In this instance, studying weather conditions leads to achieving the weather invariant perception and creating a research community to address the shortcomings. There are various seasonal influences on vision and perception everywhere on the earth or, at the very least, varied day and night circumstances in the periodic cycle. It is common for a sensor to be unable to identify lane markings, road markings, landmarks, roadside units, traffic signs, and signals in bad weather. Investigating the potential weather variation for the sensors to attain dependable autonomy is inevitable, since environmental awareness for vehicular operation is crucial.

### 1.1. Object Detection

Among the typical sensors, a camera is necessary for perceiving cars, people walking around, and, most importantly, environmental items with colors and signs, such as traffic lights and their colors, traffic signals, road signs, and instructions for driving. However, the camera is less likely to function consistently under varying weather circumstances. Another notable sensor for the contemporary self-driving car is LiDAR (light detection and ranging), which is growing in popularity due to cost reductions brought on by technological advancements. However, it is still expensive and susceptible to weather conditions such as snow or smoke. Conversely, radar (radio detection and ranging) is more dependable, because it is less affected by bad weather [2]. In addition, consistent perception may also benefit from an IMU (inertial measurement unit), a GNSS (global navigation satellite system), and ultrasonic sensors. Fusing some of the above sensors is a popular strategy for enhancing perception. Sensor fusion is quite helpful in the event of a sensor malfunctioning, particularly in severe weather. Among notable works, ref. [3] proposed an adverse weather dataset (DENSE) using various sensors containing a camera, LiDAR, radar, gated NIR (near-infrared), and FIR (far-infrared) data in fog, snow, and rain conditions to improve object detection results. Sensor fusion also enhanced detection outcomes in [4,5]. In order to find lanes in bad weather, ref. [4] used a GPS (global positioning system), LiDAR, and camera data. Ref. [5] combined complimentary LiDAR and radar data using the multimodal vehicle detection network (MVDNet). The performances and difficulties of various sensors in various weather conditions were covered in more detail through a systematic literature review in [6].

Deep learning approaches are replacing traditional perception tasks, such as object detection, tracking, etc., with newer, more potent ones as a result of the development of machine learning and AI (artificial intelligence) technologies. Deep learning frameworks were utilized by [7] to detect vehicles in foggy conditions. Ref. [7] used an attention module to better concentrate on prospective information during feature extraction. Ref. [8] offered the ZUT (Zachodniopomorski Uniwersytet Technologiczny) dataset and employed well-known YOLOv3 [9] techniques to identify pedestrians in adverse weather conditions, including rain, fog, frost, etc. There are further examples of the YOLO (you only look once) approach being used to detect pedestrians. For example, ref. [10] used YOLOv3 (and a modified version of it) to detect pedestrians in hazy weather, while [11] used the YOLO method in regular weather after enhancing the YOLOv2 model to YOLO-R for greater accuracy. Ref. [12] proposed Gaussian-YOLOv3 by reformatting the loss function of the YOLOv3 and additionally predicting the localization uncertainty of the bounding box during object detection. After applying these techniques, the detection results improved by increasing the true positives and reducing the false positives. Ref. [13] used simulated datasets derived from computer simulators to discuss the impact of different weather conditions on sensor data and its impact on obstacle detection.

Both traditional computer vision techniques and deep-learning-based frameworks are highly helpful in improving camera-based perception based on images or videos. Image enhancement, restoration, and dehazing are among the methods that may be used for images or videos to boost vision quality, and they are particularly helpful for enhancing object detection. Ref. [14] proposed the dual subnet network (DSNet) trained in an end-to-end manner and jointly learned three tasks—visibility enhancement, object classification, and object localization—to execute object detection. Ref. [15] proposed an adaptive image enhancement model called the DENet, which was trained using a neural network in an end-to-end manner and added with the YOLOv3 method to obtain DE-YOLO, which improved the detection result compared to the YOLOv3 method. Ref. [16] trained the DriveRetinex in an end-to-end manner containing two subnets, namely, Decom-Net and Enhance-Net, for decomposing a color image into a reflectance map and an illumination map; they then enhanced the light level in the illumination map. The image-enhancing network improved the object detection results trained on the Low-Light Drive (LOL-Drive) dataset collected by the authors. The image adaptive YOLO (IA-YOLO), which combines the YOLOv3 and parameter-predicted convolutional neural network known as CNN-PP, is another image-enhancing method covered in [17]. The IA-YOLO was trained end-to-end and improved the detection performance in foggy weather and low-light scenarios. By dehazing the scene and training a neural network according to their respective settings, refs. [18,19] worked on improving detection performance in hazy weather. Ref. [18] trained ReViewNet using a hybrid weighted loss function and looked twice over the hazy images to optimize the dehazing algorithm. The dehazing algorithm was used in [19] by training the BAD-Net in an end-to-end manner that connected the dehazing module and the detection module. The work also discussed the effects of image restoration results and did not use the image restoration results during the training of the networks. According to [20], as demonstrated by [17], the standard image restoration procedure was ineffective in enhancing the detection outcomes. However, the authors improved the detection performance using image restoration in cloudy and hazy conditions by concentrating on pertinent adversarial attacks [20].

The performance of deep learning frameworks may now be easily improved by increasing the volume of datasets through the arrival of new machines with increased processing power and better storage capacity. The problem of data shortage could be solved by image augmentation, data association, etc., as every neural network method relies on vast amounts of data. These data-growing strategies are becoming more popular for addressing issues such as poor detection performance. Ref. [21] presents an overall survey on vehicle perception and asserts that the corresponding research community still needs to improve vehicular perception, such as object detection in poor weather, and data fusion could solve this problem. Ref. [22] used dataset construction based on the GANs (generative adversarial networks) and cycleGANs architectures that helped to create seven versions of different weather conditions of a single dataset, and they created another seven versions of an augmented dataset from that single dataset. These datasets were produced using applicable computer techniques, such as adding fictitious droplets. The approach solved the difficulty of collecting data from the real world. It helped to learn different weather features from versions proposed in the datasets, thus improving detection results in various weather conditions. Ref. [23] also included artificial droplets to examine the performance in low-light weather augmentation for several racing car track conditions using real-world and simulator data. The detection performance was tested on various weather images containing late afternoon, sunset, dusk, night, and some different sizes of droplets. However, instead of visual accuracy, the effort mainly concentrated on the real-time performance of the perception subsystem.

Thus far, the discussions above show that several efforts have been made to enhance object detection through various methodologies. Growing data volume through various processes, such as image augmentation, artificial data creation, etc., is one of the more effective methods among them. Recent research on existing autonomous driving datasets and their covered weather aspects for perception was presented in a part of the survey in [2]. According to Table 6 proposed in [2], there are still a few research gaps, such as combining all the different types of harsh weather. As an illustration, consider integrating the three types of weather—snow, night light, and strong daylight—into a single dataset. Following the abovementioned article, our research aims to combine most weather features and analyze how feature combinations from different datasets affect object detection. Previously, in addition to the usual weather, several datasets proposed weather circumstances such as snow, rain, night light, fog, haze, smog, sand storms, clouds, overcast, etc. The work plan recommends integrating datasets from several sources containing various meteorological variables to detect autonomous-vehicle-related objects in a weather-consistent manner. This study will aid in determining the effects of feature accumulation from various datasets, diverse geographical regions, and meteorological circumstances using multiple data sources.

### 1.2. Relevant Datasets

Deep learning has recently performed outstandingly well in various visual tasks, including scene perception, object identification, object tracking, image segmentation, 3D geometry estimation, and plenty more activities that apply to autonomous vehicles. One of the best examples (pioneering work) of a deep neural network and dataset proposed for visual identification is ImageNet [24]. Similarly, various other datasets have been suggested over the last decade to enhance visual recognition results. Suppose we concentrate more intently on the usefulness of comparable type datasets in autonomous driving. In that case, the KITTI [25], Microsoft COCO [26], and Cityscapes [27] datasets have significantly contributed to modifying the usefulness of visual perception in the field of autonomous vehicles. Daimler Arban Segmentation [28], Leuven [29], TUDBrussels [30], ETH [31], INRIA [32], Daimler-DB [33], NICTA [34], CVC [35], Daimler-CB [36], Caltech [37], Camvid [38], and many more notable datasets have contributed to the diversity and quantity of resources. These datasets were gathered from the real world (or created artificially) and were utilized for various tasks, including semantic segmentation, object detection, pedestrian detection, pedestrian classification, etc.

This study focuses on particular datasets with various meteorological characteristics to achieve robust perception in severe weather. Fog, rain, night, snow, and sand storms are the primary elements of harsh weather. These elements also comprise subcomponents such as haze, mist, smog, reflected night light, rainy nights, dust tornados, rain storms, sand storms, overcast weather, clouds, shadows, etc. Some datasets attempted to include various meteorological qualities but could only independently include a few distinct weather features. Therefore, we cannot accept that those datasets are perfect for perceptions that are unaffected by any weather, nor are they generally accepted to be ubiquitously helpful for every kind of inclement weather. As a result, we selected a few valuable open datasets based on their features, utility, and weather characteristics to merge them to cover all features and create a useful repository that would eliminate any gaps in environmental characteristics. Moreover, we know that deep learning methods, i.e., neural networks, are extremely data-hungry; thus, the fusion of different datasets could be useful for learning useful features globally. There are numerous datasets for adverse weather, including Foggy Cityscape [39], LIBRE [40], CADCD [41], nuScenes [42], D2-City [43], DDD17 [44], Argoverse [45], Waymo Open [46], Snowy Driving [47], 4Seasons [48], Raincouver [49], WildDash [50], KAIST multispectral [51], EU [52], Radiate [53], etc. These datasets mostly consist of camera images from the real world (some also include GNSS, radar, LiDAR, and IMU data), thus considering various meteorological conditions from the real location. On the other hand, ALSD [54] and SYNTHIA [55] provide artificial images from simulated environments created by computers, including certain adverse weather conditions. Despite the enormous advancement in the autonomous driving data sector, we have selected a few specific datasets based on availability, features, geographical variety, and combinations of more useful weather characteristics. The online datasets discussed below (in the next paragraph) were gathered to conduct our current study. The data was gathered under the resources’ official data collection policies, and researchers were registered on their websites to request formal permission (if necessary) to utilize the resources’ data in future studies.

Since the camera is the most important sensor for environmental scene perception, particularly for traffic sign recognition, object identification, and object localization, our focus for this work has been on camera images. On the other hand, weather can also significantly affect LiDAR, but LiDAR data is out of the scope of this study. Radar and IMU can still be added as extra sensors, although doing so is optional, since inclement weather barely affects them. Among the image datasets, the Breakly Deep Drive (BDD) [56] could be a useful resource for performing the main contribution to the data merging for this work. The BDD dataset includes one hundred thousand camera images from driving footage from different American cities, including New York, Berkeley, and San Francisco. In addition to images of the usual weather, there are images of fog, rain, snow, clouds, overcast weather, and night light. Though the dataset is rich in terms of the number of images, it contains fewer images according to harsh weather features. Only 23 fog images, 213 rain images, 765 snow images, and 345 night images are useful to contribute to learning the weather features [57]. Therefore, a manual search was required to elicit those useful images from the huge dataset, which might not be feasible, and it is better to focus on a different useful dataset containing more harsh weather images and feature diversity compared to natural weather conditions. A few other datasets also suffer from similar problems or associated shortcomings, which are summarized in Table 1. Therefore, we want to choose some perfect datasets with fewer images but more intensive features from a weather characteristics standpoint; then, we could extend our study later with more normal weather images. In such a manner, the “Adverse Condition Dataset with Correspondance” dataset [57], also known as the “ACDC dataset” (https://acdc.vision.ee.ethz.ch/ (accessed on 10 October 2023)), has 4006 camera images from Zurich (Switzerland) recorded in four weather conditions: rain, fog, snow, and night. The ACDC has all photos with one of any of the weather features and 4006 images that are evenly distributed for each weather characteristic, which was very useful despite having a much smaller number of images than the BDD or Eurocity datasets. Therefore, from the perspective of usefulness, this dataset was more prosperous than the other dataset described previously. The 19 classes provided by Cityscape [27] were annotated on the ACDC dataset using pixel-level semantic segmentation and a trustworthy ground truth. This paper tested multiple existing neural networks and compared their performance outcomes on the dataset. The “Vehicle Detection in Adverse Weather Nature” dataset [58], also known as the “DAWN dataset” (https://data.mendeley.com/datasets/766ygrbt8y/3 (accessed on 10 October 2023)), which only contains 1027 photos gathered from web searches on Google and Bing, was another highly relevant dataset. However, it was selected for its extremely harsh weather qualities, which can serve as a real-world example for training and testing under adverse conditions. It also includes several sand storm images that offer distinctive aspects compared to the other datasets mentioned earlier. Seven thousand eight hundred forty-five bounding boxes for vehicles, buses, trucks, motorcycles, bicycles, pedestrians, and riders were labeled in the DAWN dataset and annotated by the LabelMe tool. The ACDC and DAWN datasets’ primary distinguishing feature includes having every image in adverse weather. Therefore, based on the above discussion, the criteria for choosing the datasets are clear now. However, we can collect them by manually selecting the relevant images from the abovementioned datasets, which might be time-consuming but relevant to extend this work further. However, we discovered that the ACDC and DAWN datasets were the most helpful for our analysis.

### 1.3. Current Study

**Figure 1:**
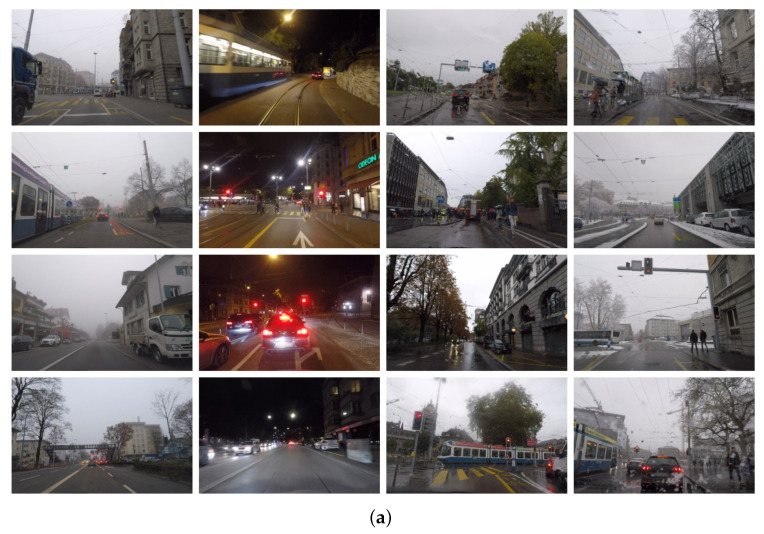

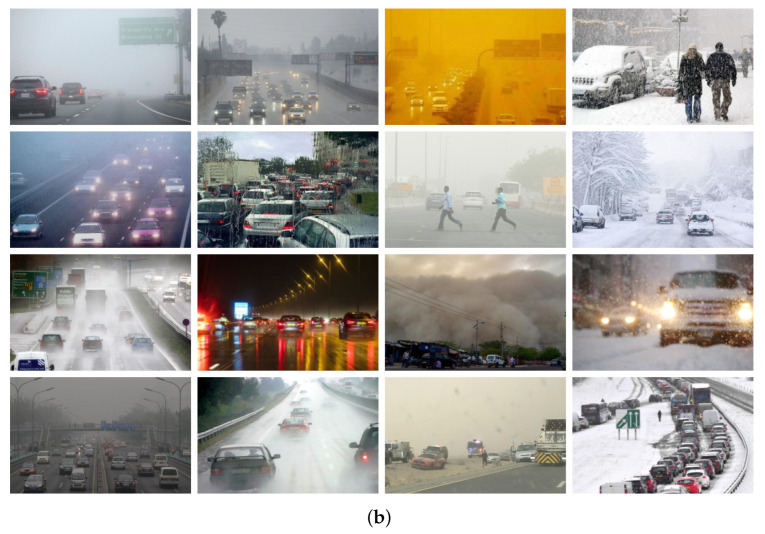
Example images from the ACDC and DAWN datasets. (**a**) Fog, night, rain, and snow images from the ACDC dataset (columnwise, respectively). (**b**) Fog, rain, sand, and snow images from the DAWN dataset (columnwise, respectively).

From the explanation above, we have concluded that the dataset and its components are numerous. Nevertheless, every dataset was annotated according to their requirements and failed to cover all of the aspects of weather conditions from a unique study. Therefore, choosing a few helpful images and merging them to cover all of the weather features in a single dataset for feeding into a neural network was an acquisitive aim. Additionally, we combined the ACDC and DAWN datasets (some example images are presented in Figure 1), which practically cover all adverse conditions, excluding direct sunshine, i.e., sun glare. Still, the unique and accurate annotation for the combined dataset was the biggest hurdle for this work. It was unable to combine the labeling, since the various datasets utilize various methods to annotate their datasets. The goal was to develop a quick and efficient approach for annotating the combined data as per the needs of object detection. Before that, knowing each dataset’s labeling procedure and strength for network training was useful for subsequent research.

Since this investigation was limited to 2D object detection through image data from camera sensors, the primary proposition of this work was to use the YOLO as an object detection method. As discussed above, a few researchers have used the YOLO method for object detection in autonomous driving. Some have tried to modify the method to boost performance (e.g., IA-YOLO, YOLO-R, DE-YOLO, Gaussian-YOLOv3, etc.). Refs. [64,65] discussed the network architectures, challenges, advantages, applications, and many more for different versions of the YOLO method. Recently, ref. [66] published the most updated information about the YOLO algorithm and discussed all the YOLO version releases until the most recent version (YOLOv8, [67]). Previously, ref. [68] performed work on object detection in bad weather for autonomous driving, but the work has several limitations. Ref. [68] suggested training a custom model using the YOLOv5 to detect objects in adverse weather. Just one model was trained in 18 min and 12 s using 239 images downloaded from the Roboflow website, and it reached an accuracy of about 25%. Comparatively, our work proposed assessing the feature merging between two datasets to open up additional prospects for combining more datasets, and, in some cases, we achieved a more than 90% accuracy utilizing the YOLOv8.

The following list of contributions (stated below) have been made by this work (ours) in its entirety: a review of the literature and discussion of various object detection techniques related to autonomous vehicles; the selection of specific datasets from a small number of relevant datasets; and the study’s overall plan, as stated in Section 1. Data collection, annotation, merging, training, and evaluation approaches are covered in the part of the methodology discussed in Section 2. A comprehensive analysis of the experiments and findings are provided in Section 3. Section 4 discussed the study’s limitations and areas for future expansion. Section 5 concludes the work by summarizing the work and providing recommendations for future researchers.

## 2. Methodology

The procedures indicated in the next subsection were used to carry out the experiment for this investigation. Following the discussion of the selection process for all relevant datasets, the collection of datasets and the process for data annotation are now briefly covered in the first subsection. Then, a description of data processing and data merging follows. Finally, the training, validating, and testing approaches are discussed with their associated evaluation criteria.

### 2.1. Data Collection and Annotation

The two datasets used for this study’s primary contribution were open-sourced and widely accessible. The DAWN dataset was primarily used for object detection in harsh weather and was annotated for six classes (car, bus, truck, motorcycle, bicycle, and person). The ACDC dataset was proposed for driving scene understanding in harsh weather through image segmentation. The ACDC dataset has been widely used for domain adaptation, such as a study of the change in the data domain. These datasets were available online, along with their corresponding annotations. However, a special labeling procedure was needed to combine them. The labels on the DAWN dataset are incompatible with the most recent versions of YOLO, even though they were created for the YOLOv3 based on Darknet architecture. The DAWN dataset, for instance, used the LabelMe tool to annotate the images, thus classifying people as label 1, bicycles as label 2, cars as label 3, and so on. Contrarily, the most recent versions of the YOLO, which were trained on the COCO dataset [26], assigns 0 to people, 1 to bicycles, 2 to cars, etc. Since transfer learning was intended to be used in this study’s custom data training, a completely new labeling order assignment is acceptable during the transfer learning. However, we followed a universal labeling process similar to the COCO dataset that is compatible with any version of YOLO trained on the COCO dataset. We planned to detect the first ten objects and keep their corresponding labels as have been defined in the COCO dataset to make unique annotations for all of the datasets we intended to merge. Namely, the annotation was defined as follows: 0—person, 1—bicycle, 2—car, 3—motorcycle, 4—airplane, 5—bus, 6—train, 7—truck, 8—boat, and 9—traffic light. Though we do not expect to detect a boat or airplane while driving on the road, we kept them as rare objects and focused more on detecting objects such as vehicles, pedestrians, traffic lights, etc. Thus, performing a new annotation was required for all images. After new labeling, the annotation is compatible with the YOLO method to use their weights as well. This study contributed to annotating both datasets to detect primary objects (the first ten objects from the COCO dataset) in harsh weather for autonomous driving. Interestingly, before this annotation, according to our knowledge, the ACDC dataset had never been labeled for object detection. The reputable data annotation website makesense.ai (https://www.makesense.ai (accessed on 10 October 2023)) assisted with the manual data annotation for this work. For the YOLO method (version 8), the makesense.ai generated labels in text format, and for the PASCAL VOC annotation (http://host.robots.ox.ac.uk/pascal/VOC/ (accessed on 10 October 2023)), labels were in HTML (hypertext markup language) format. The annotation method, which took an average of five minutes per image, attempted to incorporate all pertinent objects regardless of size and proximity to the camera. A few photos from the ACDC dataset (comparatively fewer from the DAWN) were deleted from the dataset, since they did not contain any targeted objects for detection.

### 2.2. Data Processing and Merging

After labeling all the pertinent images from two datasets, data processing was crucial in this work before training. The datasets were prepared in different versions for training, validating, and testing by the YOLOv8 algorithm. The YOLOv8 takes two different versions of image size as input for training, i.e., 640 × 640 and 1280 × 1280. Since the YOLO algorithm does not contain any image processing or augmentation process, all the image processing, including data resizing and augmentation, was performed with the help of the Roboflow website (https://roboflow.com (accessed on 10 October 2023)), which is recommended for the YOLOv8 method. The Roboflow website was useful for conducting image resizing and augmentation tasks containing horizontal flips, vertical flips, cropping, grayscales, brightness, blurring, rotations, shears, hues, saturation, exposure, noise, cutouts, mosaics, and more. The Roboflow website was also helpful in separating all images into training, validation, and testing images of 70, 20, and 10 percent ratios, respectively. Before merging the datasets, it was crucial to split them into the train, validation, and test sets, because failing to do so may result in a new combination of segregated images, thereby contaminating the evaluation dataset (test and validation images). The many combinations of relevant data augmentation techniques (crop, blur, etc.) were carried out and handled as multiple enhanced dataset versions. Through the training process, these versions assisted in evaluating the effectiveness of those versions’ findings for object detection. The relevant data augmentation mentioned here conveys that, for example, a vertical flip is not useful to this study. Nine versions of resized (640 × 640) image-augmented datasets were arranged for training. Then, the best-augmented version was chosen by the validation result during the training and selected as the final version of the augmented images used here. Additionally, the same augmentation settings also followed for the 1280 × 1280 image-sized version. Different resized versions of the same image were used because they allowed for faster training, and, occasionally, the training results revealed accuracy that was even better than the original version. Therefore, in this study, training results were presented for different versions of the processed image as follows. Here, version one contains the original dataset without augmentation or resizing, version two presents the image-augmented datasets resized by 640 × 640, version three contains the original datasets (nonaugmented) resized by 640 × 640, version four presents the original datasets resized by 1280 × 1280, and version five contains augmented datasets resized by 1280 × 1280. The five versions of the ‘MERGED’ dataset were created by merging the corresponding training, validation, and testing images from these five versions of the DAWN and ACDC datasets. According to their particular meteorological features, several merging outcomes also occurred between the DAWN and ACDC dataset subgroups. The validation and testing images were the same across the same-sized augmented or nonaugmented data versions, since the data augmentation was only performed on the training images. For a more thorough depiction, see Table 2.

### 2.3. Training and Evaluation

We employed Python programming based on the Google Colab service for training and result evaluation through validation and testing. Until this study was done, the latest version of the YOLO was the YOLOv8. This deep-learning-based neural network model is faster and gains better accuracy compared to previous versions in object detection. This study used the YOLOv8 algorithm and its pretrained weights as a backbone for training on custom data through transfer learning (https://www.analyticsvidhya.com/blog/2023/02/how-to-train-a-custom-dataset-with-yolov5/ (accessed on 10 October 2023)). As with other YOLO versions, six key attributes are responsible for object detection with a bounding box. The key attributes are the x and y coordinates of the top left corner of the bounding box, the width and height of the bounding box, a confidence score with a probability between zero and one, and a class ID. From the performance perspective, the YOLOv8 was already a perfect algorithm for object detection, and this work helped improve detection accuracy in harsh weather. The YOLOv8 GitHub repository [67] assisted in setting up training on custom data and other associated works (saving the training model, accuracy checking, etc.). Six versions of pretrained weights could be used as a base for transfer learning during training for custom data. The base weights were ‘yolov8n.pt’ for nanoobjects detection, ‘yolov8s.pt’ for small objects, and so on for medium and large objects, as well as ‘yolov8x.pt’ for extra large objects. Accuracy gained by training on these various base weights was also considered to help choose the best weight for further training.

Since all the images did not contain objects, they were removed from the respective folders of both datasets. The ACDC dataset was divided into 2715 training images, 770 validation images, and 383 testing images. Thus, only 3868 images were used among the 4006 images proposed by the original datasets. The DAWN dataset was divided into 700 training images, 203 validation images, and 100 testing images. During the accuracy evaluation, it is important to remember that the same number of images and the same images inside the validation and test sets should be present to evaluate and compare the results. Table 3 presents more details about the image distribution.

**Figure 2:**
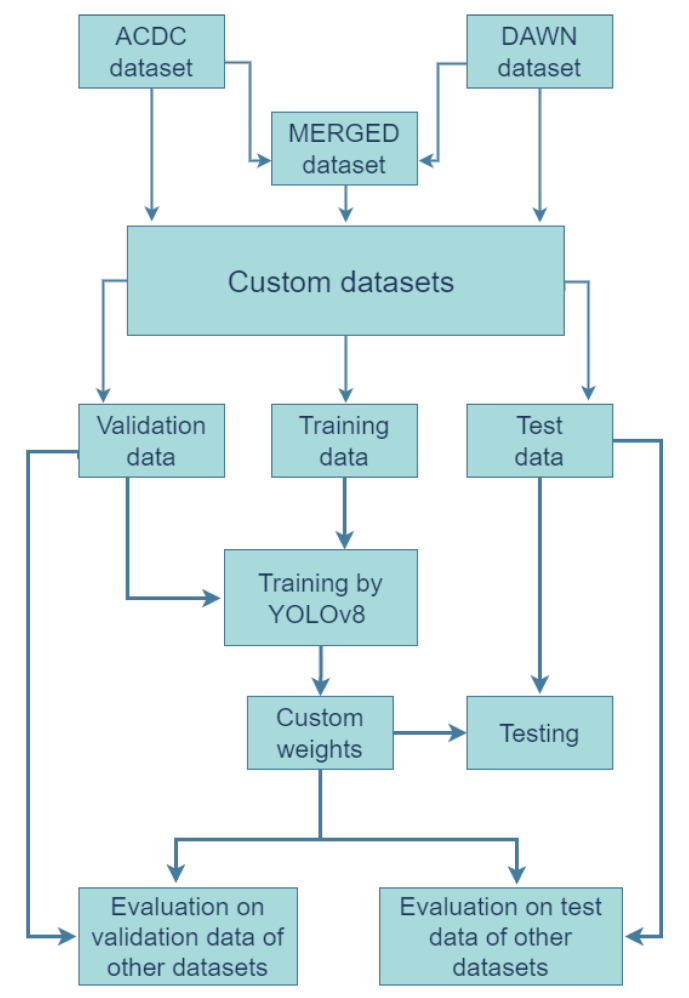
Object detection model using the YOLOv8 algorithm.

Every different version of the dataset was trained for 32 epochs (except V5) and resized to 640 × 640 during training without depending on their input data dimension. All other hyperparameters were kept at their default values for the YOLOv8 method. The training performance was evaluated on corresponding validation data after finishing the training process (result displayed after every training). These results helped us to understand the performance of the merged and associated data versions. Then, the training weights were saved, and the performance was evaluated on test sets. Thus, the weights were first performed on the corresponding validation data during training and then tested on the corresponding test set. Then, the saved weights were also performed on the test and validation sets of other data versions. The detailed workflow is shown in Figure 2.

## 3. Experiments, Results, and Discussion

All of the experiments were conducted in this work using Python under the scope of the Google Colab. The YOLOv8 algorithm helped to perform training using transfer learning on top of its base weights and custom dataset. The ACDC and DAWN datasets, as well as their merged dataset known as MERGED, were used as the custom datasets. After training, the saved weights of the different versions of training datasets were used to evaluate the performance using the various versions of the validation and test sets. The object detection results were evaluated using the mAP (mean average precision) in two different outcomes (mAP50 and mAP50-95) that were predefined by the YOLOv8 algorithm. The IoU (intersection over union) was measured as the bounding box overlap between the ground truth and predicted bounding box, and the mAP50 considered the corresponding detection as true positive where the IoU was greater than 0.5. Similarly, mAP50-95 used all different thresholds between 0.5 and 0.95 using step 0.05. This work usually used mPA50 for presenting results and rarely mAP50-95, which was only used where it was mentioned. The sequence of experiments was arranged first to choose the best base weight for training. The YOLO algorithm does not contain any image augmentation technique and assigns the augmentation part to a third party such as Roboflow. We used Roboflow to generate different augmented versions of the datasets; then, we chose the best image augmentation version based on their validation results during training. In the next step, we evaluated the training performance on the ACDC and DAWN datasets separately. We then used their merged dataset to train and evaluate the performance of the merged data and two base datasets.

The training process of the YOLOv8 can take any version of images independent of size, but it converts the training images into 640 × 640 or 1280 × 1280 pixels before feeding them into the training network. However, resizing them earlier before feeding them into the network is useful to save training time. As mentioned before, this work studied five versions: raw images (without resizing), two resized versions (1280 × 1280 and 640 × 640), and their augmented versions—which were named Raw, 1280, 640, augmented 1280, and augmented 640 (see Table 2). All five versions were used as inputs but were resized to 640 × 640 during training. We intended to train on the 1280 × 1280 version in addition to the smaller version, but the Google Colaboratory failed to manage the bigger version of the images during training due to GPU power limitations. Thus, there is a research gap in using a powerful computer to train the dataset with an image size of 1280 during training, in addition to inputs of the same size. Nevertheless, we used the five versions as inputs, despite being resized to 640 by the algorithm during training, to compare their performance outcomes and their training times.

### 3.1. Choosing the Best Weights and Augmentation

**Figure 3:**
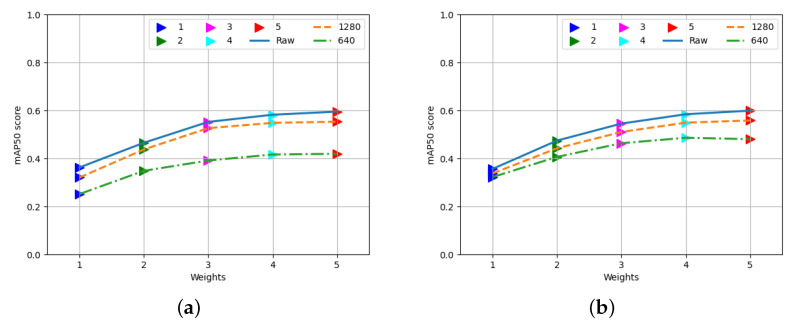
Performance of YOLOv8’s default weights on valid and test images of the ‘MERGED’ dataset. (**a**) Performance on the validation images. (**b**) Performance on the test images.

Figure 3 shows the object detection (mAP score) performance of YOLOv8’s existing weights on the validation and test sets of the MERGED images. The weights from number one to five (along the x axis) refer to the base weights for detecting nano (yolov8n.pt), small (yolov8s.pt), medium (yolov8m.pt), large (yolov8l.pt), and extra-large (yolov8x.pt) objects, respectively. The three versions of the MERGED dataset (raw and two resized versions, since the test and validation images were the same for the augmented and nonaugmented data) are presented to evaluate the detection performance of the based weights on the MERGED dataset. From the figure, it is clear that the extra-large weight performed the best object detection result on any version of images without depending on image sizes. The large and medium weights also performed well compared to the nano and small versions of the YOLOv8’s weight. It is worth mentioning that all of the weights performed below 0.6 at the mAP50 score. Figure 3 helped us choose the best base weight to train the custom datasets further via transfer learning. According to the result, we chose the extra-large weight (yolov8x.pt) as the base for the raw images, 1280 × 1280-sized images, and their augmented versions. Contrarily, the large weight (yolov8l.pt) was used for the 640 × 640-sized images and their augmented version as input images.

**Figure 4:**
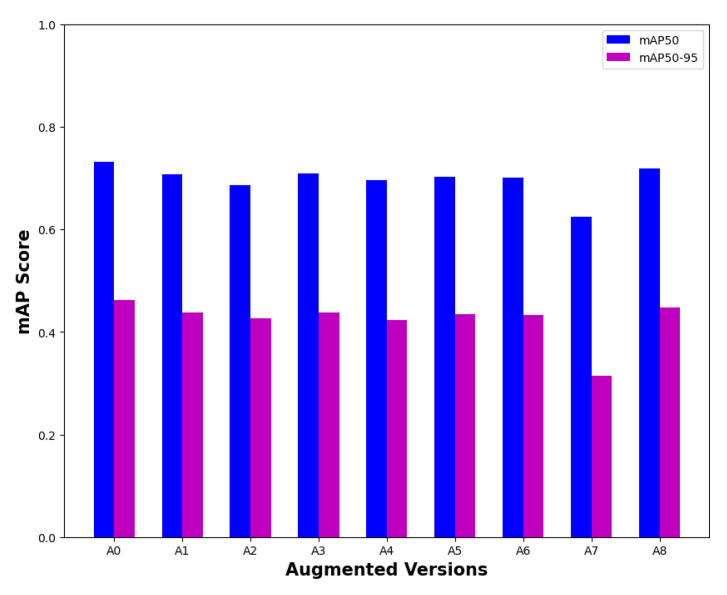
Detection performance outcomes of different versions of image augmentation.

Figure 4 presents the performance outcomes of the object detection on the validation set of the ACDC dataset by different versions of image augmentation of the ACDC dataset. First, nine different sets of augmented images were arranged from the ACDC train images and trained to evaluate the performance outcomes of the corresponding augmented versions on the ACDC validation images. Since the results are almost similar for all of the versions, we have briefly mentioned the corresponding augmentation properties used by each version in Table 4. Notably, version zero, denoted as A0, was defined as having no augmentation at all. On the other hand, version A7 contained all of the types of augmentation together, which used horizontal flips, cropping (0 to 20% zoom), rotations (−15 to 15 degrees), shears (up to 15 degrees in both horizontal and vertical directions), and all of the following by up to 25% (grayscale, hue, saturation, exposure, blurring, and brightness (both darkening and brightening)). Thus, having all of the augmentation together was not useful for better performance; even version A0 (without augmentation) performed better than any augmentation for the ACDC dataset. After examining the performance outcomes of various image augmentations, we decided to use version A8 as the augmented version for the other datasets, where horizontal flips and cropping were used as the augmentation tools.

### 3.2. Evaluation of Weights Training on the DAWN, ACDC, and MERGED Datasets

**Figure 5:**
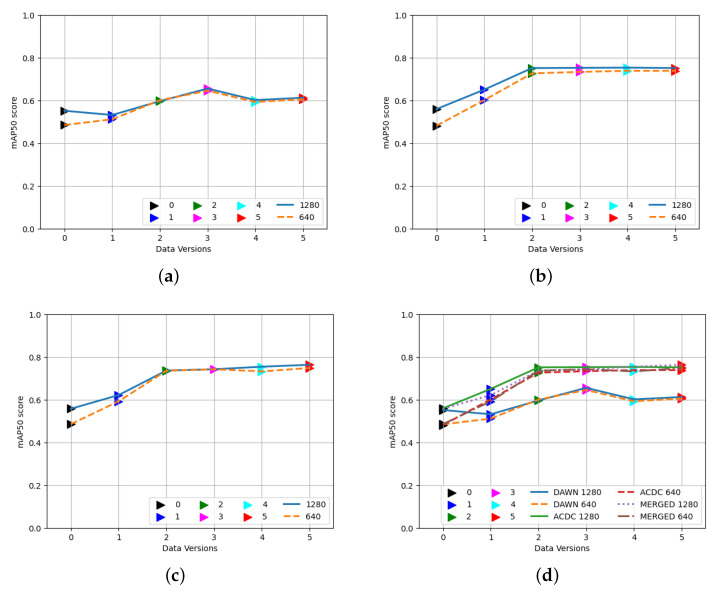
Performance of training weights using the DAWN, ACDC, and MERGED datasets on their corresponding test images. (**a**) DAWN. (**b**) ACDC. (**c**) MERGED. (**d**) All together.

Figure 5 displays the detection performance outcomes of the training weights of five different versions: the Figure 5a DAWN, Figure 5b ACDC, and Figure 5c MERGED datasets on two different sizes of test images of the corresponding dataset; Figure 5d presents all three results together. Version zero (black) mentioned here displays the performance of the YOLO algorithm’s base weights. The other nodes presented were trained on raw images (the original dataset as version one) in blue, augmented 640 (version two) in green, not augmented 640 (version three) in magenta, not augmented 1280 (version four) in cyan, and augmented 1280 (version five) in red.

From all of the first three Figure 5a–c, it is clear that training on the corresponding dataset improved the results of detection compared to the base weights of the YOLOv8 method. Even training on the resized images performed well compared to the raw images. Since the algorithm resized the training images into 640 × 640 in size during the training, independent of input sizes, the effects of different sizes of input images were almost the same for both small and large versions, even for augmented and nonaugmented versions, but they were exceptionally better than training on raw images. The bigger images as a test set (1280, presented in navy and solid lines) performed slightly better than the smaller images (640, presented in orange and dashed lines) during testing. Therefore, based on this result, we chose the best size (1280 × 1280) for evaluating valid and test images. Since the weights were produced using resized images during training, it is preferable to resize images before utilizing them for evaluation and subsequent use, because different datasets may contain images of different sizes.

Finally, Figure 5d shows all three of the results together for comparing the training performance outcomes of the mentioned weights on the corresponding dataset. The training on the ACDC dataset was better at detecting objects in its test images than the DAWN dataset. This figure also helped us to identify the accuracy elevation by training on custom data, which was uplifted to near 0.8 from below 0.6. The MERGED dataset contains more images than the ACDC dataset, and the ACDC contains more images than the DAWN. We can conclude that the number of feature-related (images containing objects we intend to detect) unique images in the training data was the reason for better performance when the merged data performed better on every subset, which we found later in the following results.

### 3.3. Effects of the MERGED Dataset on the DAWN and ACDC Dataset

**Figure 6:**
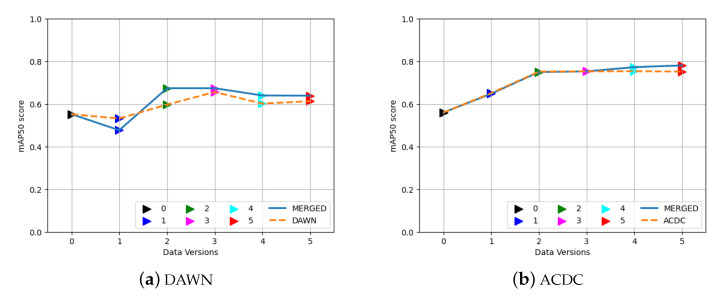
Performance of training on the MERGED dataset, (**a**) the DAWN dataset on DAWN test images, and (**b**) the ACDC dataset on ACDC test images.

Figure 6 shows the elevation in the detection performance after merging the two datasets compared to the corresponding single dataset on the test set of the single dataset. The result is the mAP50 score of the different versions of training weights collected from the different versions of the MERGED dataset and compared to the training weights of the individual dataset. Both weights were performed on the test images of the individual datasets. First, Figure 6a compares the results trained on the DAWN (dashed line) with the merged dataset (MERGED) (solid line) over the DAWN test images. Similarly, Figure 6b presents the comparison between the training weights of the ADCD dataset (dashed line) and the MERGED dataset (solid line) over the ACDC test images. Since the ACDC dataset contains approximately four times more images, the results of the DAWN dataset benefited more from the merged dataset in their detection results. As shown in Figure 6b, the performance outcomes of the weights were almost similar except for the version five input, so adding the DAWN dataset had less of an affect on improving the detection of the ACDC images. However, Figure 6a shows a significant elevation in performance after adding the ACDC dataset compared to only the DAWN dataset. Therefore, adding more images or merging more datasets could improve detection results further. Additionally, as shown in Figure 6a, the training on the raw dataset (version one) showed an impressive result: merging different datasets of various sizes could harm training results. Additionally, pre-resized inputs improved the results for both the datasets and their merged versions. However, resizing them before training also saved training time compared to the non-resized version (Table 2).

**Figure 7:**
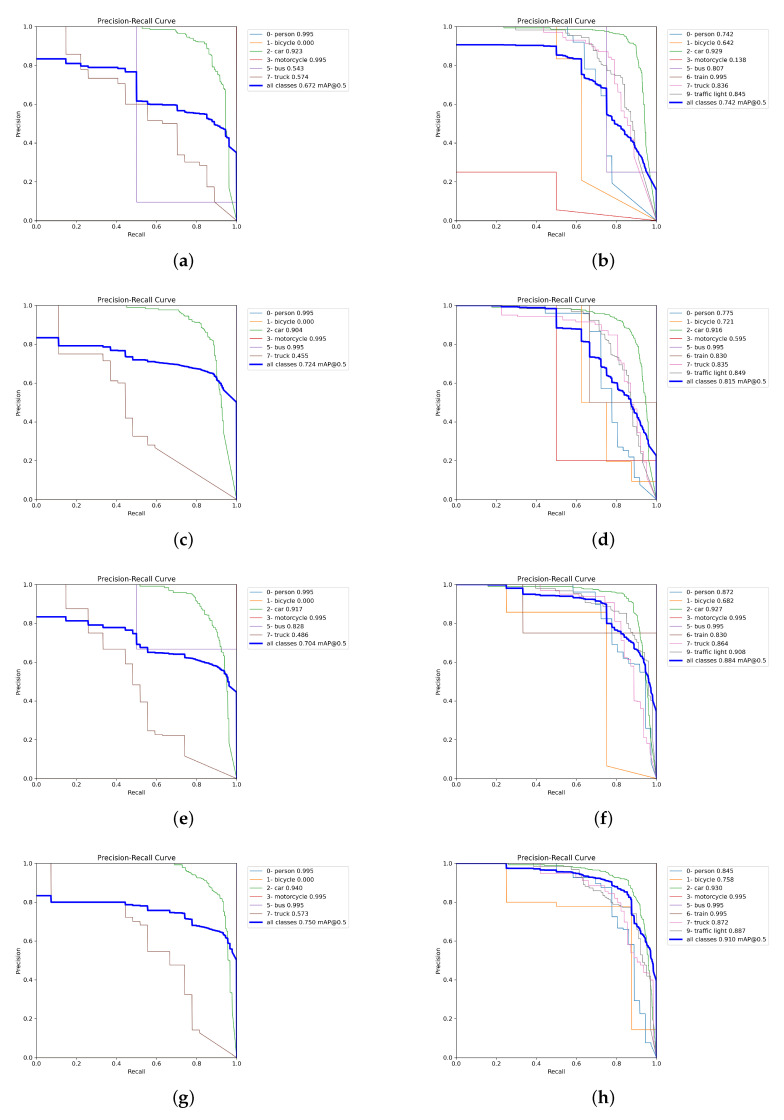
Precision–recall (PR) curves of various training weights on the fog test data. (**a**) “DAWN fog” on “DAWN fog” test data. (**b**) “ACDC fog” on “ACDC fog” test data. (**c**) “merged fog” on “DAWN fog” test data. (**d**) “merged fog” on “ACDC fog” test data. (**e**) “DAWN” on “DAWN fog” test data. (**f**) “ACDC” on “ACDC fog” test data. (**g**) “MERGED” on “DAWN fog” test data. (**h**) “MERGED” on “ACDC fog” test data.

Figure 7 presents the precision–recall (PR) curves using the training weights of all of the merged images (MERGED) (in Figure 7g,h), which were trained on the individual DAWN and ACDC (in Figure 7e,f), only merged between the fog images of the ACDC and DAWN (in Figure 7c,d), and trained on the corresponding fog images of the DAWN in Figure 7a and the ACDC in Figure 7b. The PR curves were evaluated only on the fog test images, where the left column presents the PR curves of the DAWN dataset, and the right column presents those of the ACDC dataset. From the left column, Figure 7a presents the mAP50 score of 0.672 evaluated on the DAWN fog test data using weight training on the DAWN fog data. In Figure 7c, the detection result improved for the weight training on the merged fog (merged between both the ACDC fog and DAWN fog) by up to 0.724; finally, the detection results improved to 0.75 for the weight training on the MERGED training images (in Figure 7g). However, the detection result performed by weight training on the DAWN dataset scored 0.704 (in Figure 7e), which is comparatively lower, but this case is rare and is still congruent with the relative results. Nevertheless, in the right column, the detection performed by training on the ACDC fog images gained a mAP50 score of 0.742 on the ACDC fog test images (in Figure 7b), which was improved to 0.815 for the weight training on the merged fog images (in Figure 7d). The results further improved to 0.884 for the weight training on the ACDC dataset (in Figure 7f) and boosted to a mAP50 score of 0.91 for the weight training on all of the merged (MERGED) training images (in Figure 7h). Therefore, object detection results in fog significantly improved by adding more images (feature-related) from different datasets and weather conditions.

**Figure 8:**
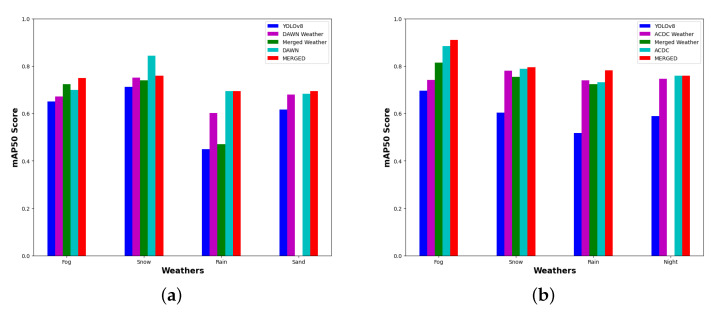
Gradual improvement of the object detection results by incorporating more images through the feature-related data merging technique. (**a**) DAWN. (**b**) ACDC.

Similarly, we have two more common weather features between the ACDC and DAWN datasets, such as rain and snow. Additionally, this included two individual weather features such as sand images for the DAWN and night images for the ACDC dataset only. We are skipping the discussion part of PR curves for those weather conditions to avoid redundancy. Instead, we have used Figure 8 to compare the mAP50 scores of the different weather and data versions. The results show a comparison between training weights’ detection scores on test images of individual weather characteristics separated into two parts for the DAWN and ACDC datasets. Each weather characteristic of the ACDC graph shows a comparison between the detection performance of the corresponding base weights and the performance outcomes trained on the “ACDC weather”, “merged weather”, “ACDC”, and “MERGED” data (in Figure 8b). The results are shown in a similar way for the DAWN dataset as well (in Figure 8a). Weight training on the MERGED dataset mentioned here implicates training on all of the merged training images. In contrast, “merged weather” means merging only the corresponding weather conditions between the two datasets. Similarly, the “DAWN weather” or “ACDC weather” mean “DAWN fog”, “ACDC rain”, etc., depending on the corresponding weather labels presented through the x axis.

The bar graphs (Figure 8) depict a common pattern of improving detection scores by adding more feature-related images. A steady increase in the detection score was clearly revealed for every trained weight compared to the base result (shown in blue). The results shown in purple represent the weights training on particular weather conditions from the corresponding dataset, which improved in performance compared to the base weights and, in some cases, performed better than the merged weather (shown in green). The weights trained on the merged weather are missing for both the sand and night images due to the fact that they are not present in both datasets together. Though they (green bar) improved the results for the fog images compared to the individual weather conditions, they did not perform well for the rain and snow images. These are examples of a few exceptions in this study, which will be investigated later through the limitation part (Section 4). The weights trained on the ACDC and DAWN datasets are depicted by cyan bars to show the improvement in detection compared to the weights trained on the data subsets (merged or individual weather conditions) discussed previously. Finally, the results using weight training on the MERGED dataset portrayed in red outperformed every result trained on the previously discussed data subsets.

**Figure 9:**
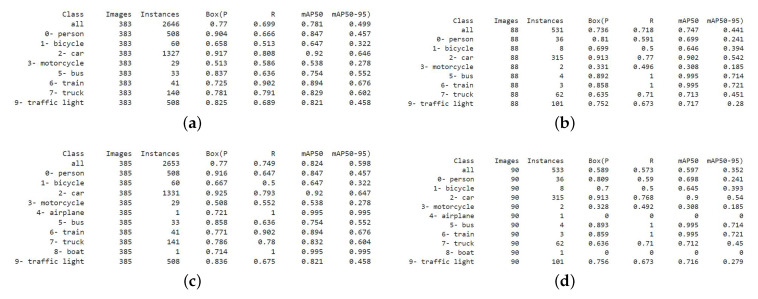
Limitations of accuracy. After adding two images, accuracy elevated between (**a**,**c**) and dropped between (**b**,**d**).

However, there are a few exceptions, such as for the DAWN rain images, where the detection result dropped unexpectedly for the weight trained on the merged rain dataset. This can be explained through feature redundancy, i.e., sometimes adding features can harm some particularly learned features of a small subset due to feature redundancy. However, the cases are rare, or else Figure 9 of the limitation part of this study (Section 4) could explain more. Still, despite a few sudden rises or falls appearing in the results, Figure 8 is evidence enough to show the gradual improvement of the object detection result by incorporating more images through the feature-related data merging technique. Finally, we have presented a few detection results (on test images) performed by the trained weights (ours) and compared them with the detection performance outcomes of the various weights of the YOLOv8 methods in Appendix A.

## 4. Limitations and Future Works

This study was conducted under the limitation of the free use of Google Colaboratory. Due to the time limitation of the GPU used by Google Colab, every training session performed here used the same number of epochs to compare their accuracy. The regular epoch size of every training was 32, but the augmented 1280 (version five) used 16 epochs for training. This study also intended to use the bigger version (image size 1280 × 1280) as the input and then use the same size during training as well. However, the bigger images were resized to 640 × 640 by the YOLO algorithm during training. Due to the GPU power limitation, it could not execute as the 1280 × 1280 image size during training. Thus, there is a research gap in improving the accuracy by training on the bigger version with powerful computational opportunities.

Another limitation of this study comes from the datasets and their annotation format. Both datasets were annotated from scratch to make them compatible together and with the YOLO method. The YOLO algorithm (version 8) was trained on the COCO dataset as the backbone, and the annotation of this study used the first ten objects in the same order. Therefore, although the DAWN images were annotated in the YOLO format for fewer objects with different annotation orders, this study required manual annotations to correct the order and to add a few more objects to detect. Thus, this study is limited to comparison with the previous results studied by other researchers. Nevertheless, an extension of this study can now use the current annotation (https://github.com/DebasisKumar21/Labels.git (accessed on 10 October 2023)) for the DAWN and ACDC datasets to improve the accuracy, which is also compatible with the YOLO weights, but adding more useful features and datasets requires compatible annotation. The annotation of this study tried to include all of the objects, even very small objects present in the far distance, to facilitate the early detection of objects. This can potentially help an autonomous system with an early warning about the presence of objects in harsh weather (see Appendix A). Thus, the accuracy might be lower compared to a different study that uses a new annotation. Another thing to mention is that the ACDC dataset was collected in Zurich, where the bus, train, and tram look similar, so it is confusing to learn a perfect feature for differentiating them. However, during the annotation of the ACDC images, the ‘Tram’ was treated as a ‘Train’ here. By considering these limitations of the annotation process, an extension of the current study is possible using the annotation files shared in the GitHub repository (Section 4) as a base and adding more relevant datasets to improve the detection results further.

Figure 9 shows a weakness in the resulting computation metric of the YOLO algorithm. Figure 9a,c show an accuracy difference by contaminating the datasets with just two images. This study used only eight objects to detect among the first ten objects of the COCO dataset, i.e., datasets were not trained to detect a boat or an airplane. Still, their place in the object list was assigned even though no instance was present in the dataset. The contamination of the dataset by two images that contain at least a boat and an airplane in the training and testing sets (using the same images so as to be detected) improved the result unexpectedly by detecting those objects. Only seven instances were added from the two additional images from Figure 9a,c. The detection results for all of the other objects were the same, but the results improved from 0.781 to 0.824 by detecting a boat and an airplane. Contrarily, from Figure 9b,d, two instances were added using two additional images (increasing 531 objects to 533). The weight failed to detect two instances (a boat and an airplane), since no boat or airplane was present in the training datasets. The result fell from a mAP50 score of 0.747 to 0.597 because of the failure to detect those particular two objects. For this study (ours), there were approximately 6996 objects present in the validation images and 3492 in the testing images. Therefore, manipulating the accuracy table by removing or contaminating images with rare objects is easy. However, this study used a fixed number of images and the same images for every test and validation set to perform the comparison fairly.

## 5. Conclusions

In this study, we proposed the use of combined data from several severe weather datasets for training through transfer learning to enhance YOLOv8-based object detection in bad weather. We used two effective open-source datasets (DAWN and ACDC) to identify important roadside objects in severe weather. First, the datasets were collected from the corresponding websites and annotated in the YOLOv8 format for the first ten objects of the COCO datasets. The datasets contain weather features of fog, rain, snow, night, and sand, and the individual weather images were divided into 70% training images, 20% validation images, and 10% testing images. Then, they were merged to separately combine the training, validation, and testing images to create a MERGED data version. Various data augmentations were also used to choose the best-augmented version according to their detection performance outcomes. The images were resized to various versions to check their performance outcomes and training times. These data versions were used to train custom weights and test their object detection performance outcomes on the testing images. The performance outcomes on the validation images were also achieved after finishing the training process, thus resulting in the accuracy table produced by the YOLO algorithm.

The proposed data merging technique improved the object detection accuracy significantly compared to the performance of the base weights of the YOLOv8 algorithm. The results compared the performance outcomes of weights training on the individual DAWN and ACDC datasets, their merged dataset (MERGED), and their distinct weather subsets. The results presented via graphs (Figure 6) show that the MERGED dataset performed better than the weights training on the individual datasets. The accuracy improvement presented in the bar graphs (Figure 8) shows that training on a custom dataset improves the object detection results further, and the accuracy was elevated with the addition of more images (with relevant data features). Noticeably, the training weight collected from training on the MERGED datasets performed best on every subset of the relevant dataset, thereby gradually becoming better than training on a particular subset after merging those subsets. Thus, this study concludes that merging more diverse images of feature-relevant datasets could perform better for object detection.

These findings provide the following research-related insights. The detection outcomes could be enhanced even more by starting with the base datasets and labels presented here and adding more datasets to them. A more powerful computer may further enhance the outcomes by using larger image sizes during training or by training for more epochs. The meteorological features utilized here cover nearly every adverse weather situation, but some environmental factors, such as sun glare, are still absent. In addition to severe weather, more images from datasets with regular weather could also lead to more accurate object detection.

## Figures and Tables

**Table 1:** Usefulness and shortcomings of some relevant datasets.

Datasets	BDD [56]	Eurocity [59]	Mapillary [60]	P.F.B. [61]	ApolloScape [62]	GLARE [63]
**No. of images**	100,000	47,300	25,000	254,064	143,906	2157
**Pros**	Huge number of images collected from various cities of USA.	Gathered from 31 cities across 12 European countries.	The most diverse dataset in the geographical context.	Huge number of images.	Contains sun glare images.	Contains sun glare images.
**Cons**	Lower number of harsh weather images.	Mainly focused on pedestrian detection.	Lower number of harsh weather images.	Lower number of harsh weather images (artificial).	Lower number of harsh weather images.	For traffic sign detection only.

**Table 2:** Explanation of training, validation, and testing data versions.

Weights Trained on					Weights Evaluated on	
**Weight Trained on Version No.**	**Input Images**	**Base Weights**	**Size of Images during Training**	**Approximate Training Time for the MERGED Dataset (32 Epochs)**	**Size of Validation Images**	**Size of Test Images**
V1	Raw images	yolov8x.pt	640 × 640	4 h	Raw images	Raw images
V2	640 × 640 (augmented)	yolov8l.pt	640 × 640	5 h	640 × 640	640 × 640
V3	640 × 640	yolov8l.pt	640 × 640	2 h	640 × 640	640 × 640
V4	1280 × 1280	yolov8x.pt	640 × 640	3.5 h	1280 × 1280	1280 × 1280
V5	1280 × 1280 (augmented)	yolov8x.pt	640 × 640	4.5 h for 16 epochs	1280 × 1280	1280 × 1280

**Table 3:** Image distribution.

	DAWN				ACDC				MERGED			
**Weather**	**Train**	**Valid**	**Test**	**Total**	**Train**	**Valid**	**Test**	**Total**	**Train**	**Valid**	**Test**	**Total**
Sand	223	63	33	319	0	0	0	0	223	63	33	319
Fog	193	59	27	279	638	179	88	905	831	238	115	1184
Rain	142	40	19	201	698	198	98	994	840	238	117	1195
Snow	142	41	21	204	700	200	100	1000	842	241	121	1204
Night	0	0	0	0	679	193	97	969	679	193	97	969
Total	700	203	100	1003	2715	770	383	3868	3415	973	483	4871

**Table 4:** Used properties of augmentation in this study. “All*” mentioned here incorporates all types of augmentation explained (with their properties) in the corresponding section. “No*” conveys no augmentation.

Versions	A0	A1	A2	A3	A4	A5	A6	A7	A8
**Types of Augmentation**	No*	Flip, Crop, Rotation	Flip, Crop, Rotation, Shear, Blur	Flip, Crop, Rotation, Grayscale	Flip, Crop, Rotation, Hue, Saturation	Flip, Crop, Rotation, Exposure, Brightness	Flip, Crop, Rotation, Hue, Brightness	All*	Flip, Crop

**Figure A1:**
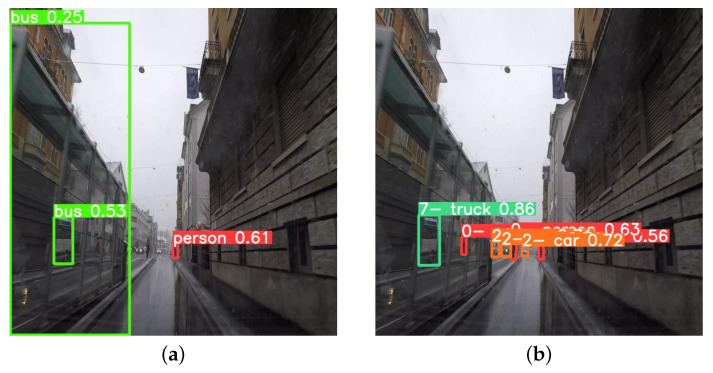
Performance outcomes of YOLOv8’s base weight (‘yolov8x.pt’) (in left column) and weight trained on MERGED dataset (V5) (in right column), on the test images (ACDC, DAWN). (**a**) Detection by ‘yolov8x.pt’. (**b**) Detection by ‘MERGEDv5.pt’. Different colors are used for different types of objects (0—person (red), 2—car (orange), 5—bus (light green), 7—truck (green)).

**Figure A2:**
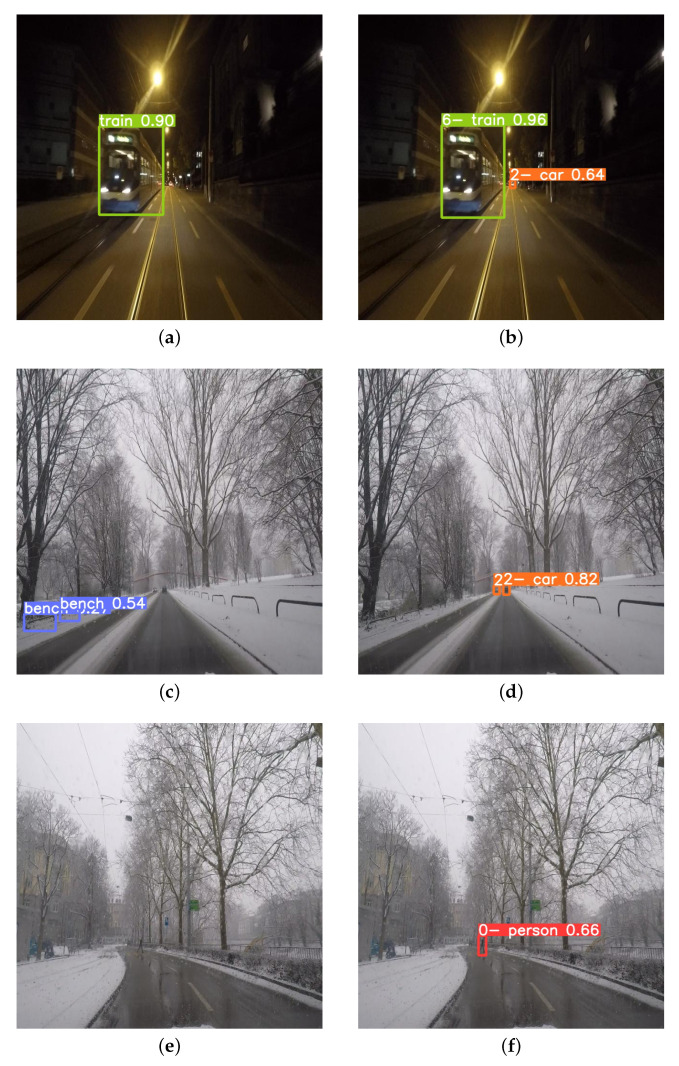
Performance outcomes of YOLOv8’s base weight (‘yolov8x.pt’) (in left column) and weight trained on MERGED dataset (V5) (in right column) on the test images (ACDC, DAWN). Row-wise comparison (on a pair of images) between detection performance outcomes of both the weights. (**a**) Detection by ‘yolov8x.pt’. (**b**) Detection by ‘MERGEDv5.pt’. (**c**) Detection by ‘yolov8x.pt’. (**d**) Detection by ‘MERGEDv5.pt’. (**e**) Detection by ‘yolov8x.pt’. (**f**) Detection by ‘MERGEDv5.pt’.

**Figure A3:**
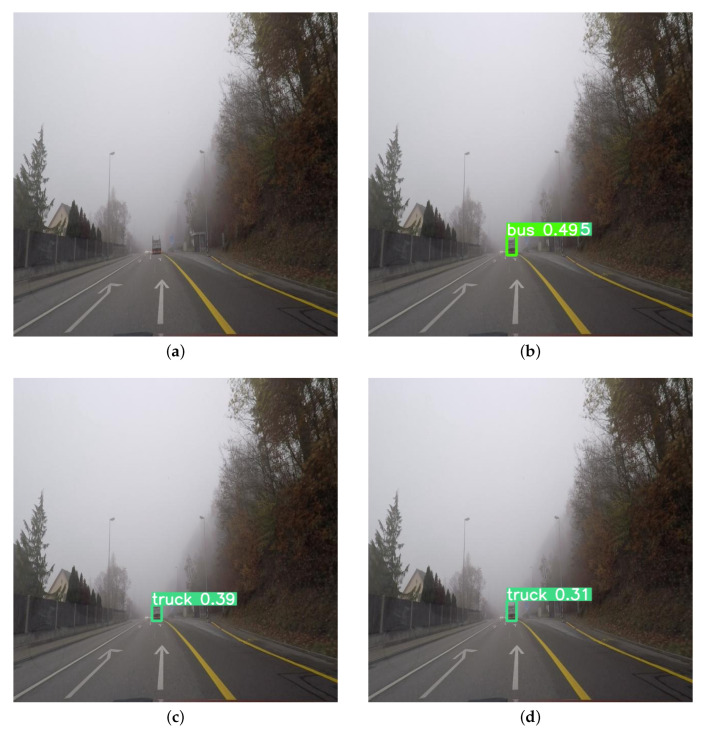

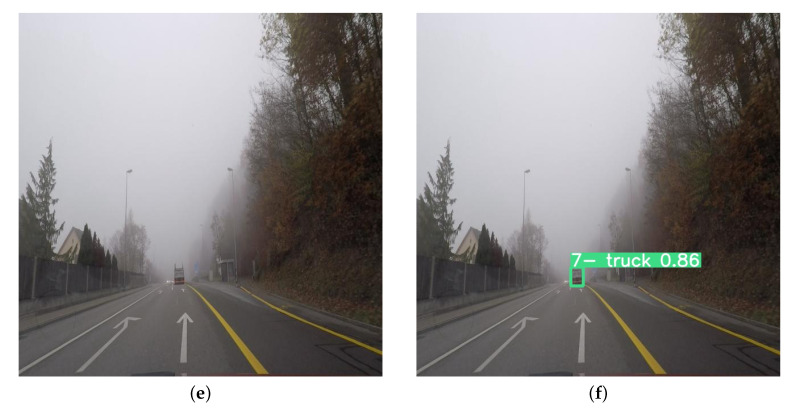
Performance outcomes of YOLOv8’s base weights and weight trained on MERGED dataset (V5) on the test images (ACDC, DAWN). (**a**) Detection by ‘yolov8n.pt’. (**b**) Detection by ‘yolov8s.pt’. (**c**) Detection by ‘yolov8m.pt’. (**d**) Detection by ‘yolov8l.pt’. (**e**) Detection by ‘yolov8x.pt’. (**f**) Detection by ‘MERGEDv5.pt’.

**Figure A4:**
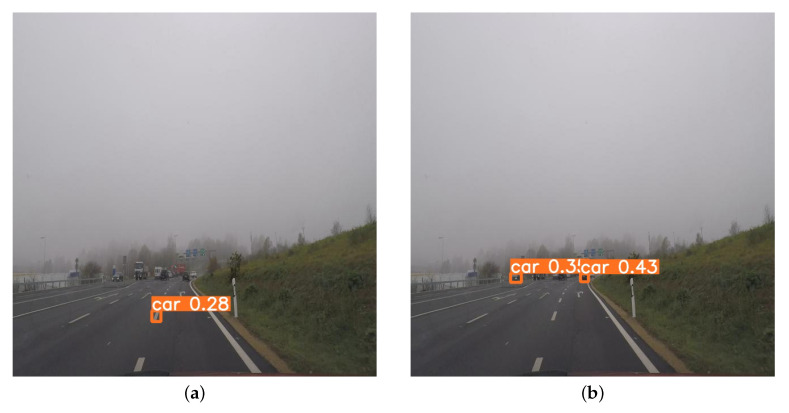

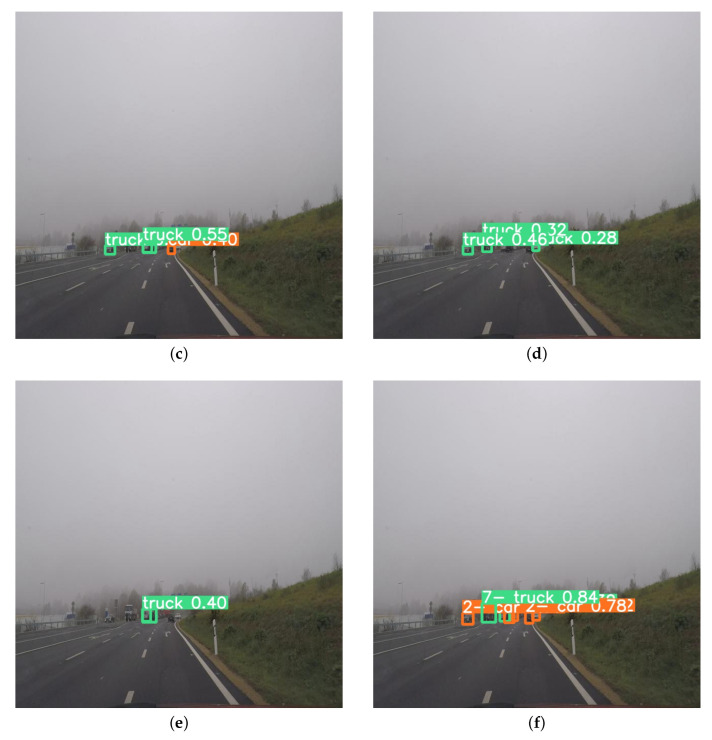
Performance outcomes of YOLOv8’s base weights and weight trained on MERGED dataset (V5) on the test images (ACDC, DAWN). Different colors are used for different types of objects (2—car (orange), 7—truck (green)). (**a**) Detection by ‘yolov8n.pt’. (**b**) Detection by ‘yolov8s.pt’. (**c**) Detection by ‘yolov8m.pt’. (**d**) Detection by ‘yolov8l.pt’. (**e**) Detection by ‘yolov8x.pt’. (**f**) Detection by ‘MERGEDv5.pt’.

**Figure A5:**
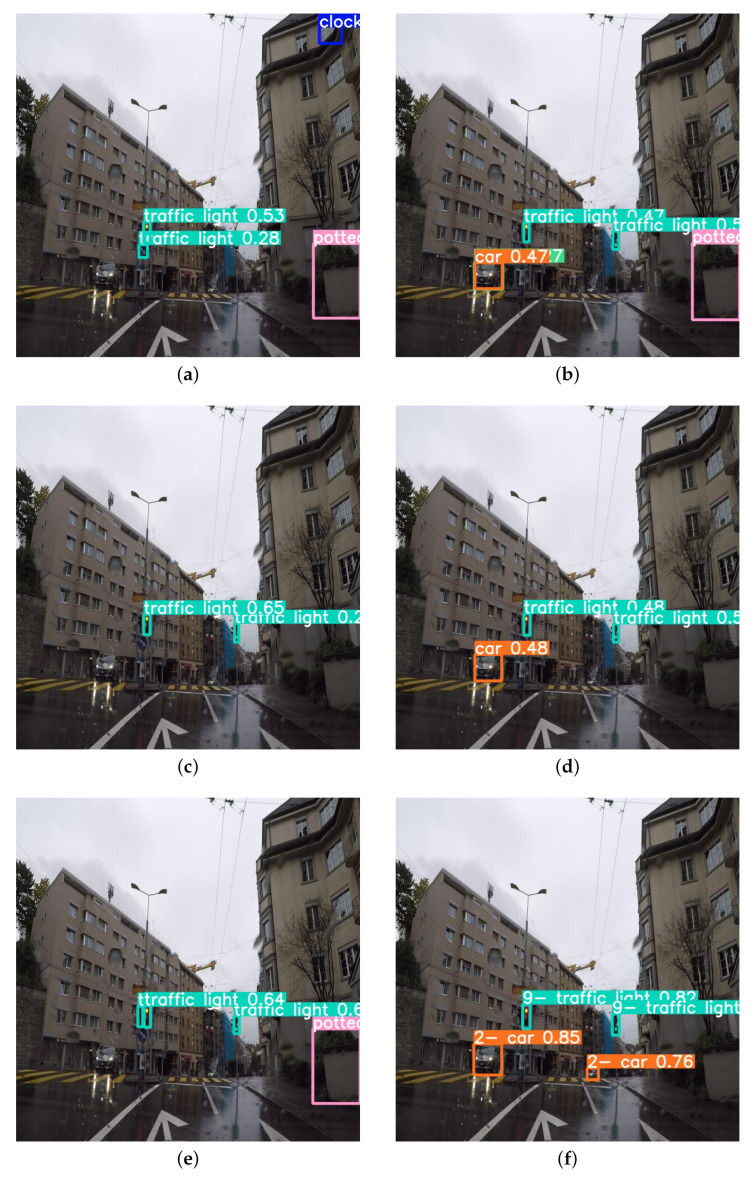
Performance outcomes of YOLOv8’s base weights and weight trained on MERGED dataset (V5) on the test images (ACDC, DAWN). (**a**) Detection by ‘yolov8n.pt’. (**b**) Detection by ‘yolov8s.pt’. (**c**) Detection by ‘yolov8m.pt’. (**d**) Detection by ‘yolov8l.pt’. (**e**) Detection by ‘yolov8x.pt’. (**f**) Detection by ‘MERGEDv5.pt’.

**Figure A6:**
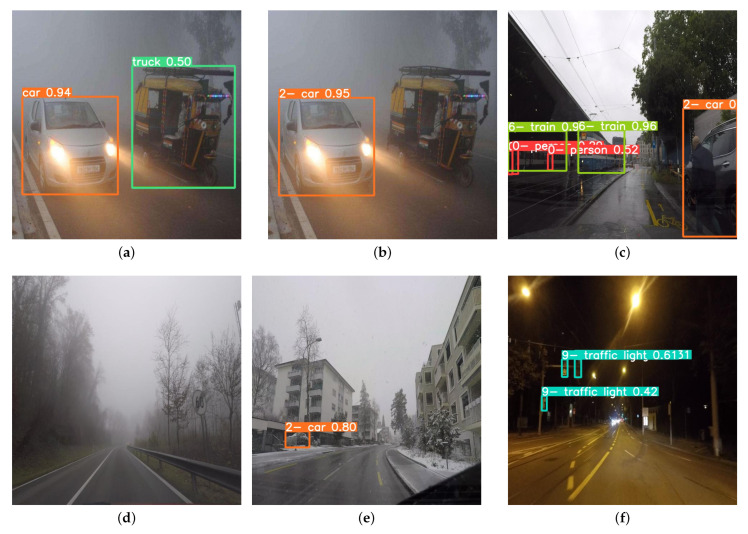
Some missed detections (test images from the ACDC and DAWN datasets) by our trained weight (MERGEDv5.pt) (except (**a**) that is detected by ‘yolov8x.pt’). Different colors are used for different types of objects (0—person (red), 2—car (orange), 6—train (olive), 7—truck (green), 9—traffic light (ocean blue)).

## Data Availability

All data are available from the corresponding author upon request.

## Appendix A

In this appendix section, we have shown a few comparisons between the detection results of our trained weights and various versions of YOLOv8’s base weights. We performed detection of the weights on test images (from the ACDC and DAWN datasets) and chose the few most significant among them to show the results.

First, we chose the weight (MERGEDv5.pt) of the MERGED dataset (V5) as the trained weight, since that version performed better on the detection we discussed earlier. Moreover, among YOLOv8’s base weights, we chose ‘yolov8x.pt’ to show the performance of the YOLOv8 method. Then, we extended the detection results by incorporating other base weights provided by the YOLOv8 method. As shown in Figure A1, the detection was performed on a rain image by YOLOv8 and our trained weight, which are (a) and (b), respectively. It is clear from both of the images that the trained weight detected all of the relevant objects successfully, while YOLOv8 missed two ‘persons’, and three ‘cars’, detected one ‘truck’ as a ‘bus’, and, most importantly, mistakenly detected a ‘passenger canopies’ as a ‘bus’ (Figure A1a).

Figure A2 shows the detection performance of YOLOv8 (in the left column) and the trained weight (in the right column) on the same row-wise images. Both Figure A2a,b show a ‘train’ detected successfully, but, as shown in Figure A2a, (YOLOv8) failed to detect a ‘car’ that appeared far from the camera. Thus, our trained weight is capable of detecting those small objects that are far from the camera to aid in early detection in harsh weather. Similarly, Figure A2c (YOLOv8) shows a misidentification compared to Figure A2d (ours) for detecting two ‘cars’ that appeared far away in snowy weather. Figure A2e (YOLOv8) also failed to identify a ‘person’ in the snowy weather. However, our trained weight successfully detected the person shown in Figure A2f. These early detections of small objects (independent of the distance from the ego vehicle), besides detecting other relevant bigger objects (which are easy to detect), can help to prevent accidents by warning about the proximity of those small objects.

From the above discussion, we have seen that our trained weight is better at detecting relevant objects independent of sizes and proximity, and it is less confused about the object’s identity. Since YOLOv8 contains other base weights to detect various objects depending on their sizes (as discussed earlier), we tried to compare our results with those versions as well. Figure A3 shows the detection results (on a foggy image) of the five base weights (YOLOv8) and the weight (MERGEDv5.pt) trained on the MERGED dataset (V5). Our weight detected a ‘truck’ with a confident score of 0.86 (Figure A3f), while ‘yolov8m.pt’ and ‘yolov8l.pt’ detected with a confident score of less than 0.4 (Figure A3c,d, respectively); ‘yolov8n.pt’ and ‘yolov8x.pt’ failed to detect the object (Figure A3a,e, respectively), and ‘yolov8s.pt’ detected the object as a ‘bus’ (Figure A3b). Therefore, the other versions of the base weights are also not as trustworthy as our trained weight. Similarly, as shown in Figure A4, ‘MERGEDv5.pt’ successfully detected all of the objects as two ‘trucks’, and five ‘cars’ (Figure A4f). However, all of the base weights of the YOLOv8 failed to detect those objects perfectly. Finally, we have shown another example from a rainy image in Figure A5, where all of the base weights (YOLOv8) failed to detect the second car (the smallest between the two; both were detected, as shown in Figure A5f, by the trained weight). Moreover, three of the base weights failed to detect the bigger car as well (Figure A5a,c,e).

Therefore, from this section, we can conclude that our trained weight performs better for detection (even for the smallest or farthest objects) than the weights of the YOLOv8 algorithm. The early detection performed by our work in harsh weather will contribute to autonomous vehicles being able to perceive better. Moreover, for autonomous driving to be a reality in all geographical locations worldwide, autonomy in harsh weather conditions needs to be solved, and our work addresses perception (for application in autonomous driving) from a broad range of adverse weather conditions.

Finally, Figure A6 shows some missed detections by our trained weight (MERGEDv5.pt) (except for Figure A6a). Figure A6a,b show the detection by ‘yolov8x.pt’ and ‘MERGEDv5.pt’ on a foggy image, respectively. The vehicle that our trained weight missed (Figure A6b) was detected as a ‘truck’ by YOLOv8 (Figure A6a); however, the vehicle was labeled as a ‘car’ in our test set. As shown in Figure A6c, the ‘MERGEDv5.pt’ detected the car present on the right side of the image, and it also detected the person present in front of the car, but the name (‘person’ ) and the confidence score of the person was missing, while the label (zero) was showing for the detected person. Thus, we are confused about the missed detections. However, we have tested our trained weight on all of the test images and rarely found any missed detection. Nevertheless, for a few other images (Figure A6d–f), the ‘MERGEDv5.pt’ failed to detect some vehicles that were very far from the sight of the ego vehicle, and they were so small that it is even hard to see them in the images presented here. Those vehicles could only be detected through their headlights’ feature, and we successfully detected most of those small objects except for a few, such as the ones presented here.
